# The *DcPS1* cooperates with *OSDLa* during pollen development and 2n gamete production in carnation meiosis

**DOI:** 10.1186/s12870-022-03648-z

**Published:** 2022-05-24

**Authors:** Xuhong Zhou, Shuying Li, Xiaomi Yang

**Affiliations:** 1 Office of Science and Technology, Yunnan University of Chinese Medicine, 1076 Yuhua Road, Chenggong, Kunming, Yunnan 650500 PR China; 2 Flower Research Institute, Yunnan Academy of Agricultural Sciences, National Engineering Research Center for Ornamental Horticulture, 2238 Beijing Road, Kunming, Yunnan 650205 PR China

**Keywords:** Meiosis, 2n gamete, Spindle, DcPS1, OSDLa

## Abstract

**Background:**

Deciphering the mechanisms of meiosis has important implications for potential applications in plant breeding programmes and species evolution. However, the process of meiosis is poorly understood in carnation, which is famous for its cut flowers.

**Results:**

We report that *Dianthus caryophyllus* parallel spindle 1 (*DcPS1*) regulates omission of second division like a (*OSDLa*) during pollen development and 2n gamete production in carnation meiosis. In DcPS1 and OSDLa RNAi lines, an absence of the second meiotic division and the abnormal orientation of spindles at meiosis II might be the main reason for dyad/triad formation, resulting in unreduced gametes. We also found that carnation OSDLa interacted with DcPS1 and DcRAD51D. In the DcPS1 RNAi lines, a decrease in *OSDLa* and *DcRAD51D* expression was observed. In the OSDLa RNAi lines, a decrease in *DcPS1* and *DcRAD51D* expression was also observed. We propose that *DcPS1* regulates *OSDLa* expression, allowing entry into meiosis II and the proper orientation of the metaphase II spindle in meiosis II. We also propose that *OSDLa* regulates *DcRAD51D* expression, allowing for homologous recombination.

**Conclusions:**

These results suggest a critical role for *DcPS1* and *OSDLa* in diplogamete production during meiosis and open a new pathway for meiosis-related studies.

**Supplementary Information:**

The online version contains supplementary material available at 10.1186/s12870-022-03648-z.

## Background

Polyploidy occurs when more than two complete sets of chromosomes are present in one species and is an important feature in plant species evolution. Polyploidy is widely regarded as a favourable force for evolution and species formation [1, 2]. Because chromosome sets are duplicated in polyploids, the heterozygosity may be fixed, and random mutations or factors that regulate gene expression may be buffered (unlike in a diploid), so new genes and gene functions may evolve, and the original function of genes may be maintained in the other set of chromosomes [3].

Polyploidy can occur through a variety of pathways, such as the rapid and efficient formation of unreduced gametes through parental hybridization breeding of polyploids and their offspring. Most researchers believe that sexual polyploidization leading to unreduced gametes is the main origin [4]. Fertilization involving unreduced gametes (2n) is a common source of triploid and tetraploid organisms: failure of a meiotic division or spindle orientation abnormalities result in 2n gametes [5, 6]. The formation of fertile polyploids not only promotes heredity and diversity but also facilitates polyploid breeding. Thus, 2n gametes can largely transmit parental heterozygosity traits to offspring [7]. The formation mechanism, i.e., second-division restitution (SDR) or first-division restitution (FDR), greatly affects the gametic and population structures as well as the breeding efficiency [4].

The use of unreduced gametes in plant breeding has led to the establishment of sexual polyploids and has played an important role in the improvement of crops such as carnation [8], Japanese persimmon [9], lemon [10], lily [11, 12], poplar [13], potato [14], rose [15, 16] and rubber trees [17]. Triploids with important horticultural traits, such as vigorous growth and large flowers, were identified in tulip 2x × 2x hybrid progenies [18]. Bilateral sexual polyploidization with the 2n gametes resulted in increased biomass, early flowering, large seeds, large cells and large leaves in *Medicago sativa* [19]. Importantly, economically important traits including resistance to abiotic and biotic stresses, were introgressed from wild species into cultivated potatoes through 2n gametes [20].

*Arabidopsis thaliana parallel spindle 1* (*AtPS1*) is associated with the formation of a high frequency of diplogametes in plants. Abnormal spindle orientation at male meiosis II leads to the formation of diplogametes [5]. Omission of second division 1 (*OSD1*) was also involved in 2n gamete formation. Loss of *OSD1* function was originally shown to cause defects in the second mitotic division during the meiotic cell cycle, thus resulting in the production of diploid male and female gametes in *A. thaliana* [21]. *OSD1* and its homologue *UVI4* (UV insensitive 4) are negative regulators of the anaphase-promoting complex/cyclosome (*APC/C*) [22, 23]. The mutant *osd1* has large epidermal cells in its cotyledons with higher ploidy, indicating that *OSD1* plays a role in endoreduplication or endomitosis in cotyledons [23]. The loss of *UVI4* function leads to an increase in somatic tissue resistance to UV-B and an increase in the ploidy level, which indicates that *UVI4* inhibits endocycles [24]. The ploidy indices of both the *uvi4–2* and *osd1–2* mutants were significantly higher than that of Ler-0. Therefore, the expression levels of *OSD1* and *UVI4* are the key determinants for cells to enter regular mitosis or endoreduplication [25].

Carnation is one of the most popular commercial cut flowers in the world. It is favoured by many exporting countries because of its excellent freshness, rich forms and colours, and its ability to withstand long-distance transportation. Cut carnations, roses and chrysanthemums account for nearly 50% of the world cut flower trade [26]. Additionally, 2n pollen was found in carnation, and the expression frequency was less than 5%. The frequency of diploid pollen in carnation varied across seasons and different genotypes [27].

Among the various cytological mechanisms of carnation 2n gametogenesis, the most common mechanisms are meiosis II spindle defects and the absence of meiosis II. Interestingly, *DcPS1* may play a role in male meiosis and ovary development. The decrease in *DcPS1* expression reflected the 2n pollen phenotype. There may be a correlation between the decreased expression of *DcPS1* and the frequency of 2n gametogenesis [27, 28]. In carnation, *OSDLa* is continuously expressed in buds and many other tissues, indicating its role in meiosis and somatic cell growth [28]. However, these hypotheses regarding the functions of *DcPS1* and *OSDLa* in carnation have not been confirmed. This paper focuses on the functions and the regulatory network of *DcPS1*and *OSDLa*.

## Methods

### Plant materials and growth conditions

The carnation variety ‘Nogalte’ was used in this study. Carnation shoots were taken from plants grown in a greenhouse under natural day length and temperature. Shoots were soaked in 70% ethanol for 5 seconds, surface sterilized for 20 min in 3% NaClO solution and one drop of Tween-20, and then rinsed three times at 5 min each in sterilized water. Shoots were grown on plates containing MS medium containing 1 mg/L BA and 0.1 mg/L NAA.

### Plasmid construction and plant transformation

To test for functional conservation of *DcPS1* (GenBank number KR013247) and *OSDLa* (GenBank number KX622764), a construct containing the *DcPS1*and *OSDLa* cDNA fragments fused to the CaMV 35S promoter was generated (Supplementary Fig. 1) and introduced into wild-type carnation. First, a 500-bp fragment of the *DcPS1* coding region (nucleotides 1900 to 2399) was amplified by PCR with unique XhoI and KpnI sites and cloned into PJL10. Then, the PCR-derived fragments were inserted into two regions flanked by ClaI and XbaI sites in opposite directions, the *PDK* intron linker sequence was flanked by the two inverted repeats (Supplementary Fig. 1), and the resulting plasmid was named 35S:RNAi-DcPS1. A 150 bp fragment of the *OSDLa* coding region (nucleotides 142 to 291) was ligated in sense and antisense into the PJL10 vector in the same way as the fragment of *DcPS1*, and the plasmid was named 35S:RNAi-OSDLa.

The shoot meristem of carnation was used as an explant for genetic transformation as previously described [29]. *A. tumefaciens* strain C58, which contained either the 35S:RNAi-DcPS1 or 35S:RNAi-OSDLa vector, was used to inoculate the explants. Regenerated plantlets were acclimatized and grown in a contained greenhouse. Tissue from the transgenic carnation plants was collected for further analysis and stored at − 80 °C until use.

### Screening transgenic plants by PCR

Genomic DNA was isolated from young leaves of putative transformants. Primers PDK-1262F and DcPS1 R (Supplementary Table S1) were used to amplify a fragment of the PDK intron sequence and the *DcPS1* inserted fragment for screening DcPS1 RNAi transgenic plants. The primers PDK F and OSDLa R (Supplementary Table S1) were the same as those used to amplify a fragment of the PDK intron sequence and the *OSDLa* inserted fragment for screening OSDLa RNAi transgenic plants.

### RNA isolation and RT–qPCR

Total RNA was isolated from wild-type and transgenic plants using with TRIzol reagent (TransGen, China). First-strand cDNA was synthesized from 1 μg of total RNA with Hifair™ II 1st Strand cDNA Synthesis Super Mix for qPCR (gDNA digester plus) (Yeasen, China) according to the manufacturer’s protocol and used for qPCR. A fragment of *GAPDH* was amplified as an internal control for calibrating relative levels of gene expression. QPCR was carried out using the GAPDH 579-F and GAPDH 788-R primers in the *GAPDH* gene, DcPS1–2118F and DcPS1–2225R primers in the *DcPS1* gene, OSDLa-422F and OSDLa-656R primers in the *OSDLa* gene, and DcRAD51D-530F and DcRAD51D-701R primers in the *DcRAD51D* gene (Supplementary Table S1). Data were analysed using the statistical program SPSS Software Version 18.0. All assays were performed in triplicate. Different letters indicate significant differences (*P* < 0.05).

### Cytology

Observation of final male meiotic products and chromosome spreads was carried out as previously described [27]. Anthers were removed from collected young floral buds and fixed in Carnoy’s solution at room temperature for 24 h. The anthers were excised on glass slides and stained with carbol-fuchsin for 1 hour or DAPI solution for 30 minutes in the dark at room temperature and then covered with cover glass. Observations were made under a Nikon E800 microscope at × 1000. The numbers of dyads, triads, and tetrads were counted when meiotic cells were at the tetrad stage.

### Cloning and testing bait for autoactivation

The full-length sequences of *DcPS1*, *OSDLa*, and *DcRAD51D* (GenBank number MK733915) were obtained by PCR. Primers DcPS1 F and DcPS1 R, OSDLa F and OSDLa R, and DcRAD51 F and DcRAD51 R were used to amplify fragments of the *DcPS1*, and *OSDLa* and *DcRAD51* genes, respectively (Supplementary Table S1). The fragments were inserted into pGBKT7 vectors individually. To evaluate the effect on yeast growth, pGBKT7- OSDLa, pGBKT7-DcRAD51D, pGBKT7- DcPS1 and pGBKT7 vectors were separately transformed into *S.cerevisiae* strain Y2HGold (Clontech, USA) using Yeastmaker Yeast Transformation System 2 (Clontech, USA). The transformed cells were grown in SD/−Ade-His-Leu-Trp/X-α-Gal/AbA medium at 30 °C for 3–5 days. Blue colonies appeared, which indicated self-activation of the plasmid. These assays were repeated at least three times.

### Confirmation and analysis of positive interactions

For the confirmation assays, the *OSDLa* and *DcRAD51D* coding sequences were inserted into the vectors pGADT7 and pGBKT7, respectively. The two plasmids were cotransformed into Y2HGold yeast cells to confirm their interactions. The transformants were grown in SD-Trp-Leu-His-Ade/X-α-Gal/AbA media at 30 °C for 3–5 days. Detailed procedures from the Yeast Handbook (Clontech, Japan) were followed. These assays were repeated at least three times.

### Transient expression assays in *N. benthamiana* leaves

BiFC assays were conducted as previously described [30]. Each full-length coding sequence of the *DcPS1* and *DcRAD51D* genes were fused with N-terminal *YFP* (YFP^N^), and those of *OSDLa* and *DcRAD51D* were fused with C-terminal *YFP* (YFP^C^)*.* Agrobacterium strain GV3101 was transformed with the above vector, and then the proper plasmid pairs were cotransformed and injected into young leaves of *N. benthamiana*. Plants were grown in weak light for 2 days. Images were captured using a confocal laser scanning microscope (Nikon, Japan). These assays were repeated three times.

## Results

### The DcPS1 RNAi line produces diploid male gametes in carnation

To explore *DcPS1* function in carnation, an RNAi construct was generated utilizing a 500 bp region, and this fragment was inserted into an RNAi vector. 35S:RNAi-DcPS1 was introduced into carnation calli using Agrobacterium-mediated T-DNA transfer. One independent DcPS1 RNAi line was recovered, and transgene integration was verified by PCR (Supplementary Figs. 2A, 3). The results showed that the expression of *DcPS1* was significantly decreased (t test, *P* < 0.05) in transgenic plants compared with the wild type (Supplementary Fig. 2B).

**Fig. 1:**
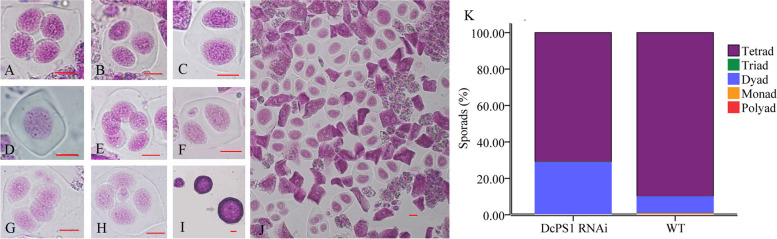
Abnormal PMCs observed at tetrad stage in WT and *DcPS1* RNAi line. **A** normal tetrad, (**B**) triad, (**C**) dyad, (**D**) monad, (**E**) pentad; (**F**) dyad with one microcyte, (**G**) tetrad with two microcyte, (**H**) tetrad with one microcyte, (**I**) 2n pollen (arrow), (**J**) a lot of dyads formation in the transgenic carnation, bar = 10 μm, (**K**) quantification of meiotic products in wild type and DcPS1 RNAi line, WT (*n* = 3302) and DcPS1 RNAi (*n* = 2489)

At the tetrad stage of pollen mother cells (PMCs) in the WT and DcPS1 RNAi lines, a total of 3302 and 2489 PMCs were observed, respectively. Among them, the vast majority (89.95%) were tetrads in WT (Fig. 1A). In addition, we found 0.18% triads (Fig. 1B), 9.18% dyads (Fig. 1C), 0.67% monads (Fig. 1D) and 0.03% polyads (Fig. 1E), as well as a few abnormal dyads (Fig. 1F) and tetrads (Fig. 1G, H) in WT. The dyads and triads eventually produced two 2n pollen grains and one 2n pollen grain (Fig. 1I), respectively. In the DcPS1 RNAi line, however, we found 71.03% tetrads, 0.24% triads, 28.6% dyads, and 0.12% monads. The production of dyads in the DcPS1 RNAi line was observed at a higher frequency than that in wild type (Fig. 1J, K). These data strongly supported the conclusion that *DcPS1* is involved in diplogamete production in carnation.

**Fig. 2:**
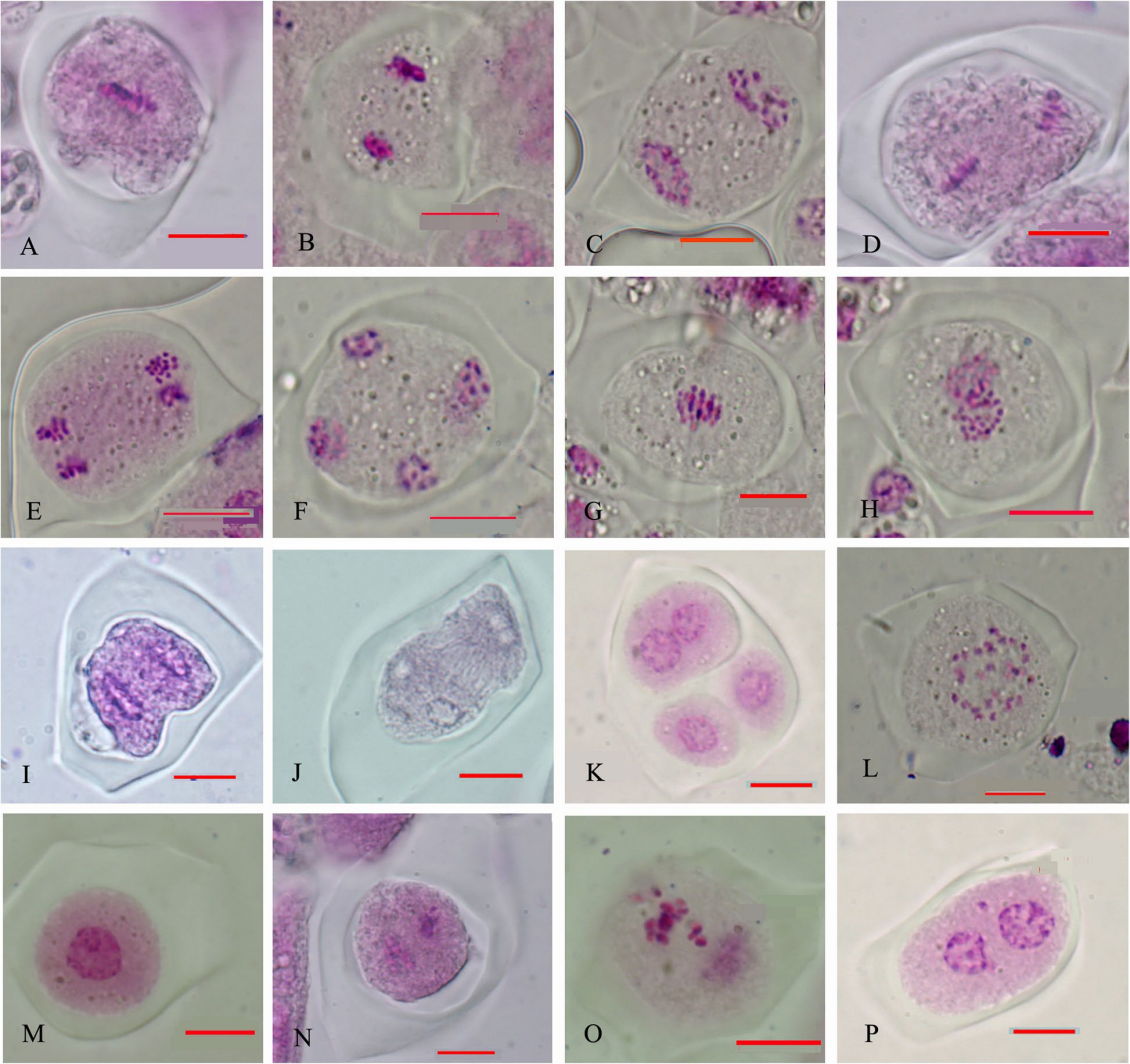
Normal (**A**-**F**) and abnormal (**G**-**P**) meiotic chromosome behaviors in microsporocytes of WT and DcPS1 RNAi line staining with carbol fuchsin. (**A**) metaphase I, (**B**) anaphase I, (**C**) telophase I, (**D**) metaphase II, (**E**) anaphase II, (**F**) telophase II, (**G**) metaphase I, (**H**) anaphase I, (**I**) aberrant cytokinesis, (**J**) tripolar spindles, (K) triad, (L) fused spindles, (M) monad, (N,O) lagged chromosomes at metaphase II, (**P**) lagged chromosomes at telophase II, bar = 10 μm

Both normal and abnormal meiosis were observed during microsporogenesis in the WT and DcPS1 RNAi lines. In normal meiosis, the metaphase I spindle aligns the bivalents at the equatorial plane (Fig. 2A) and subsequently segregates the homologues towards the opposite poles at anaphase I (Fig. 2B). Chromosomes decondensed at telophase I at the end of meiosis I (Fig. 2C). Perpendicular spindles are usually observed at metaphase II (Fig. 2D), leading to the formation of four well-separated poles at anaphase II and telophase II (Fig. 2E, F) and tetrads at the end of meiosis. In abnormal meiosis, abnormalities were observed in meiosis I and II, including an absence of the second meiotic division (Fig. 2G-I), tripolar spindles (Fig. 2J), fused spindles (Fig. 2L) and lagged chromosomes at metaphase II (Fig. 2N, O) and telophase II (Fig. 2P). These defects might be the main reason for dyad and triad formation in the DcPS1 RNAi line, which occurred at a higher frequency than in the wild type.

### The OSDLa RNAi line produces diploid male gametes in carnation

PCR amplification was used to screen positive transgenic lines (Supplementary Figs. 4A, 5). Four transgenic plants were obtained in which *OSDLa* expression was suppressed to varying degrees in different RNAi lines. The results showed that the expression of *OSDLa* was significantly decreased (t test, *P* < 0.05) in transgenic plants compared with the wild type (Supplementary Fig. 4B).

**Fig. 3:**
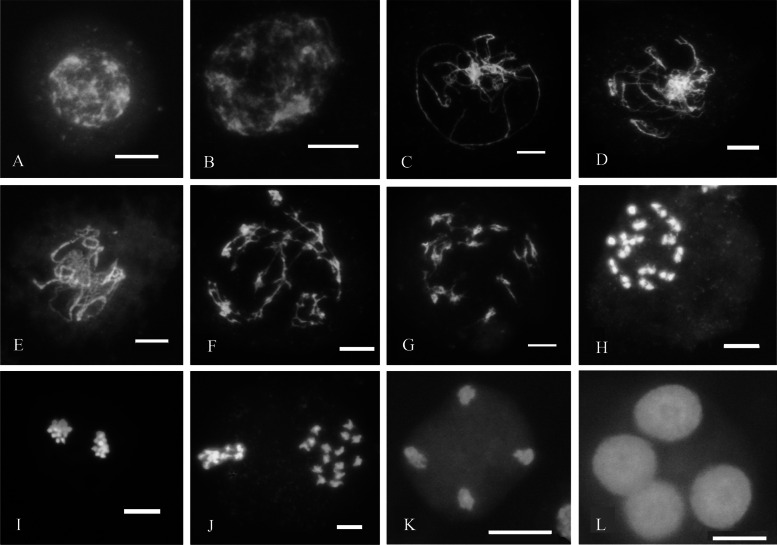
Normal meiotic cytological observation of transgenic positive plants stained with DAPI. **A**-**B** Leptotene, (**C**-**D**) Leptotene –Zygotene, (**E**) Pachytene, (**F**-**G**) Diplotene, (**H**) Metaphase I, (**I**) Anaphase I, (**J**) Metaphase II, (**K**) Anaphase II, (**L**) Tetrad. Bar = 10um

**Fig. 4:**
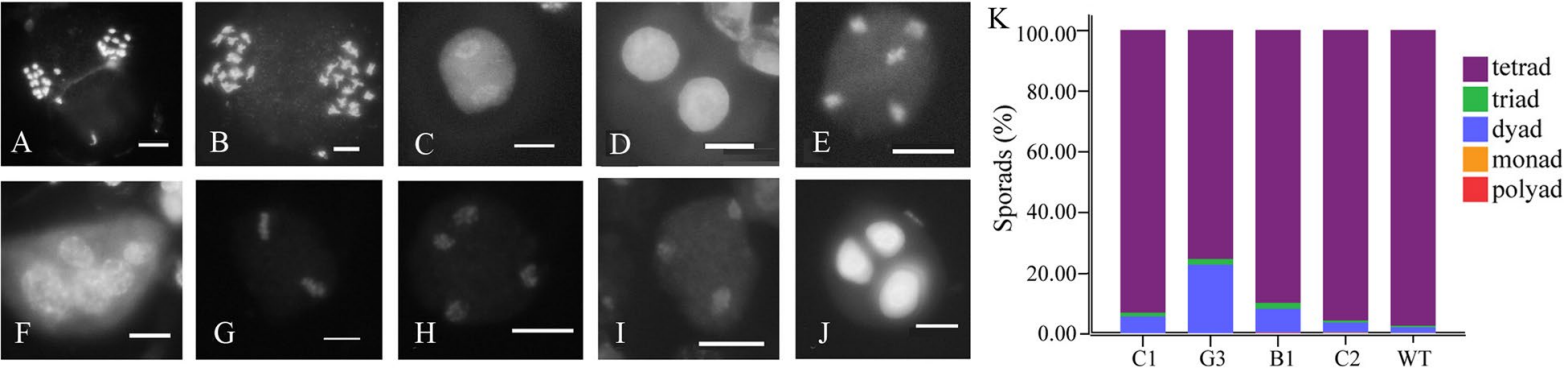
Abnormal meiotic cytological observation of OSDLa RNAi lines stained with DAPI. **A** Anaphase I, showing chromosome bridge, (**B**) Anaphase I, showing a stage of homologous chromosome separation, (**C**) Telophase I, (**D**) Dyad, (**E**) Anaphase II, showing unbalanced chromosome distribution, (**F**) unbalanced polyads, (**G**) Metaphase II, (**H**) Anaphase II, tripolar spindle, (**I**) Telophase II, (**J**) triad, Bar = 10um. (K) quantification of meiotic products in wild type and OSDLa RNAi lines, WT (n = 7360) and DcPS1 RNAi C1 (*n* = 1326), G3 (*n* = 363), B1 (*n* = 509), C2 (*n* = 4254)

The meiotic division of some microspores in transgenic carnation plants proceeded normally (Fig. 3). At the leptotene stage, the chromatin condensed into a filamentous structure due to spiral curling (Fig. 3A, B). At the zygotene stage, homologous chromosomes gradually moved closer and started pairing, and the synaptonemal complex (SC) also began to form at this stage (Fig. 3C, D). The chromosomes were further condensed and fully synapsed along the SC at pachytene, and the nonsister chromatids of homologous chromosomes recombined locally (Fig. 3E). Homologous chromosomes were further condensed, and the SC began to disintegrate and fall off chromosomes, but homologous chromosomes were still cross-linked (Fig. 3F, G). Chromosomes were neatly arranged on the metaphase I equatorial plate (Fig. 3H), and homologous chromosomes were separated and moved to the two poles at anaphase I (Fig. 3I). The two sets of fifteen homologues aligned neatly on the two metaphase II plates (Fig. 3J). The second round of segregation at anaphase II (Fig. 3K) led to the formation of four sets of chromosomes that decondensed to form the spore nuclei and finally formed a tetrad (Fig. 3L). In OSDLa RNAi line meiocytes, abnormal chromosome bridges appeared at meiosis I (Fig. 4A). Homologous chromosomes separated at telophase I (Fig. 4B) and formed a dyad (Fig. 4C, D), suggesting that dyad production is due to an absence of the second meiotic division. Lagging chromosomes at anaphase II (Fig. 4E) maybe lead to unbalanced polyads (Fig. 4F). Oriented metaphase II/anaphase II spindles were aberrant and formed tripolar spindles (Fig. 4G, H). This defect in spindle orientation explained the appearance of triads (Fig. 4I, J).

At the tetrad stage of pollen mother cells (PMCs) in WT, OSDLa RNAi plants C1, C2, G3 and B1, a total of 7360, 1326, 4254, 363 and 509 PMCs were observed, respectively. In wild-type diploid plants, 97.53% tetrads were observed (Fig. 4K). In addition, we found 0.56% triads, 1.79% dyads, 0.11% monads and 0.01% polyads (Fig. 4K). In the OSDLa RNAi plant C1, 93.21% tetrads, 1.28% triads, 5.51% dyads were observed. In the OSDLa RNAi plant C2, we found 95.79% tetrads, 0.68% triads, 3.31% dyads, 0.19% monads and 0.02% polyads. In the OSDLa RNAi plant G3, 75.48% tetrads, 1.93% triads, 22.59% dyads were found. In the OSDLa RNAi plant B1, 89.98% tetrads, 1.96% triads, 7.86% dyads and 0.2% polyads were observed (Fig. 4K). Compared to the WT, in the OSDLa RNAi lines, a high frequency of dyads and triads was observed, confirming that the diploid microspores in OSDLa RNAi lines are produced by the defects in the meiotic process.

### Carnation OSDLa interacts with DcPS1 and DcRAD51D

**Fig. 5:**
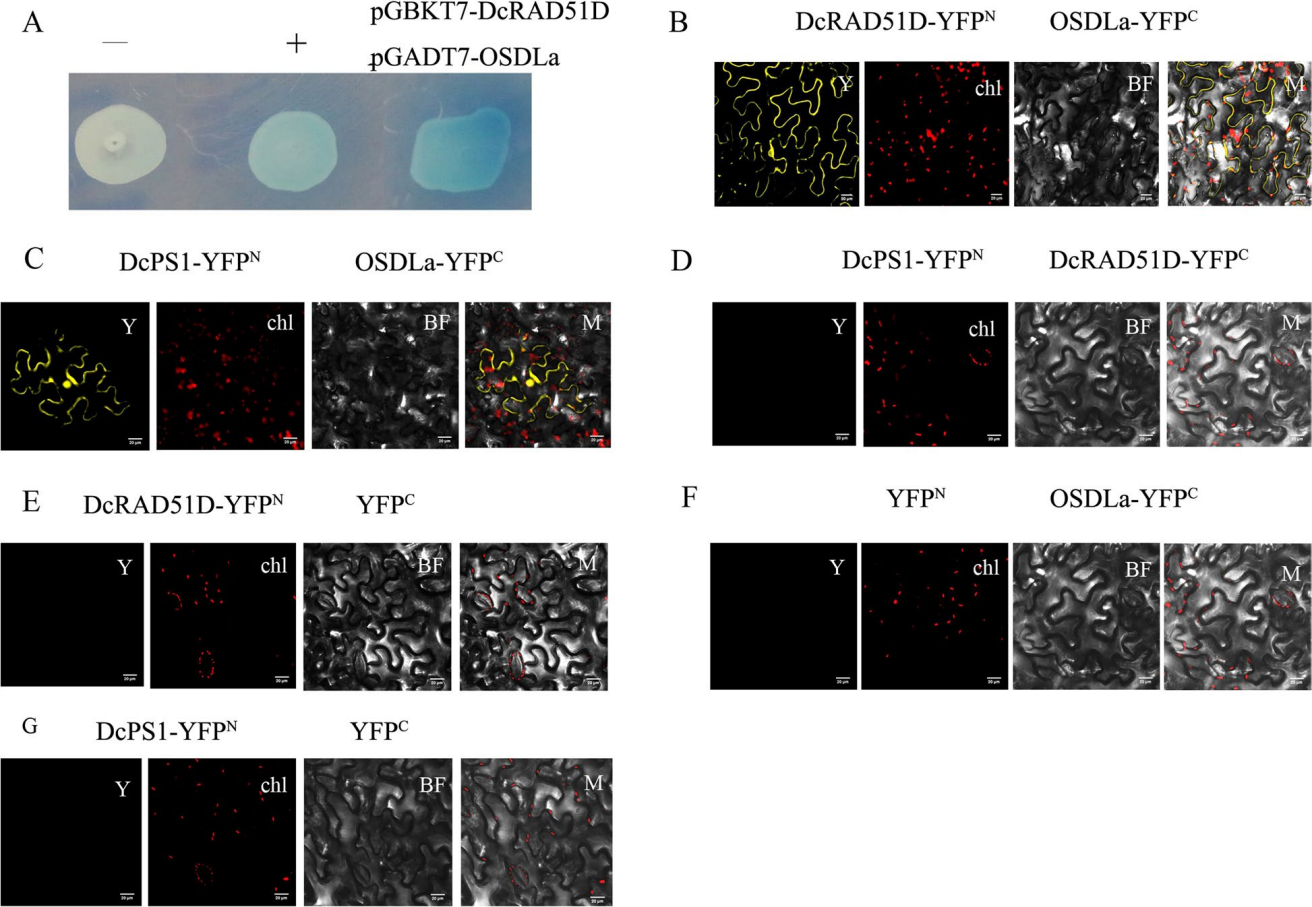
DcRAD51D and DcPS1 physically interacts with OSDLa. **A** Y2H assay for the interaction of DcRAD51D and OSDLa. Co-transformation with pGADT7-T and pGBKT7-Lam was used as a negative control, while co-transformation with pGADT7-T and pGBKT7–53 was used as a positive control. **B** BiFC analysis for in vivo interaction between DcRAD51D and OSDLa. **C** BiFC analysis for in vivo interaction between DcPS1 and OSDLa. **D** DcPS1 were unable to associate with DcRAD51D. For two combinations, DcRAD51D-YFP^N^ + YFP^C^  (Co-transformation with DcRAD51D-YFP^N^ and YFP^C^) (**E**), YFP^N^ + OSDLa-YFP^C^   (Co-transformation with YFP^N^ and OSDLa-YFP^C^) (**F**) and DcPS1-YFP^N^ + YFP^C^  (Co-transformation with DcPS1-YFP^N^ and YFP^C^) (**G**) were a negative control. Y, YFP; Chl, Chlorophyll; BF, Bright Field; M, Merge

We chose full-length *DcPS1*, *DcRAD51D* and *OSDLa* to further analyse protein-protein interactions. The vectors pGBKT7-DcPS1, pGBKT7-DcRAD51D and pGBKT7-OSDLa were transformed into the Y2HGold yeast strain, and the resulting transformants were plated on SD medium lacking tryptophan but containing a chromogenic substrate for yeast galactosidase and aureobasidin A (SD/−Trp/X-α-gal/AbA). The pGBKT7-DcPS1 colonies turned blue on SD/ -Trp/X-α-gal/AbA plates (Supplementary Fig. 6A), while the pGBKT7-DcRAD51D and pGBKT7-OSDLa colonies did not turn blue (Supplementary Fig. 6B, C). This result indicated that the bait pGBKT7-DcPS1 could autonomously activate the reporter genes,whereas pGBKT7-DcRAD51D and pGBKT7-OSDLa could not autonomously activate the reporter genes in the absence of prey protein and were, therefore, suitable for screening in the Y2H assay. Y2HGold cells were cotransformed with pGBKT7-DcRAD51D and pGADT7-OSDLa and plated on QDO/X/A plates, and a positive interaction was indicated by the presence of blue colonies (Fig. 5A). The results showed that carnation DcRAD51D interacted with OSDLa.

The DcRAD51D-OSDLa interaction was also confirmed via bimolecular fluorescence complementation (BiFC) assays in Nicotiana protoplasts in vivo, in which DcRAD51D was fused to the amino-terminal half of yellow fluorescent protein (YFP) (DcRAD51D-YFP^N^) and OSDLa was fused to the carboxy-terminal half of YFP (OSDLa-YFP^C^). For two combinations (DcRAD51D-YFP^N^ + OSDLa-YFP^C^ and DcPS1-YFP^N^ + OSDLa-YFP^C^), YFP signals were observed in the nucleus and cytoplasm (Fig. 5B, C). There was no YFP signal when DcPS1-YFP^N^ was cotransformed with DcRAD51D -YFP^C^ (Fig. 5D). These results showed that carnation OSDLa interacted with DcPS1 in vivo, and OSDLa interacted with DcRAD51D both in vivo and in vitro, whereas DcPS1 was unable to associate with DcRAD51D.

### *DcPS1* positively regulates the expression levels of *OSDLa* and *DcRAD51D*

Similar to the DcPS1 RNAi lines, RNAi lines for OSDLa also produced high numbers of unreduced microspores through the absence of the second meiotic division and a tripolar spindle mechanism at meiosis II (MII) (Fig. 4). Moreover, the frequencies of dyad/triad formation in DcPS1 and OSDLa RNAi lines were higher than those in the wild type (Figs. 1 and 4). BiFC analysis also confirmed that OSDLa interacted with DcPS1 (Fig. 5), suggesting that both genes are involved in the same molecular pathway.

**Fig. 6:**
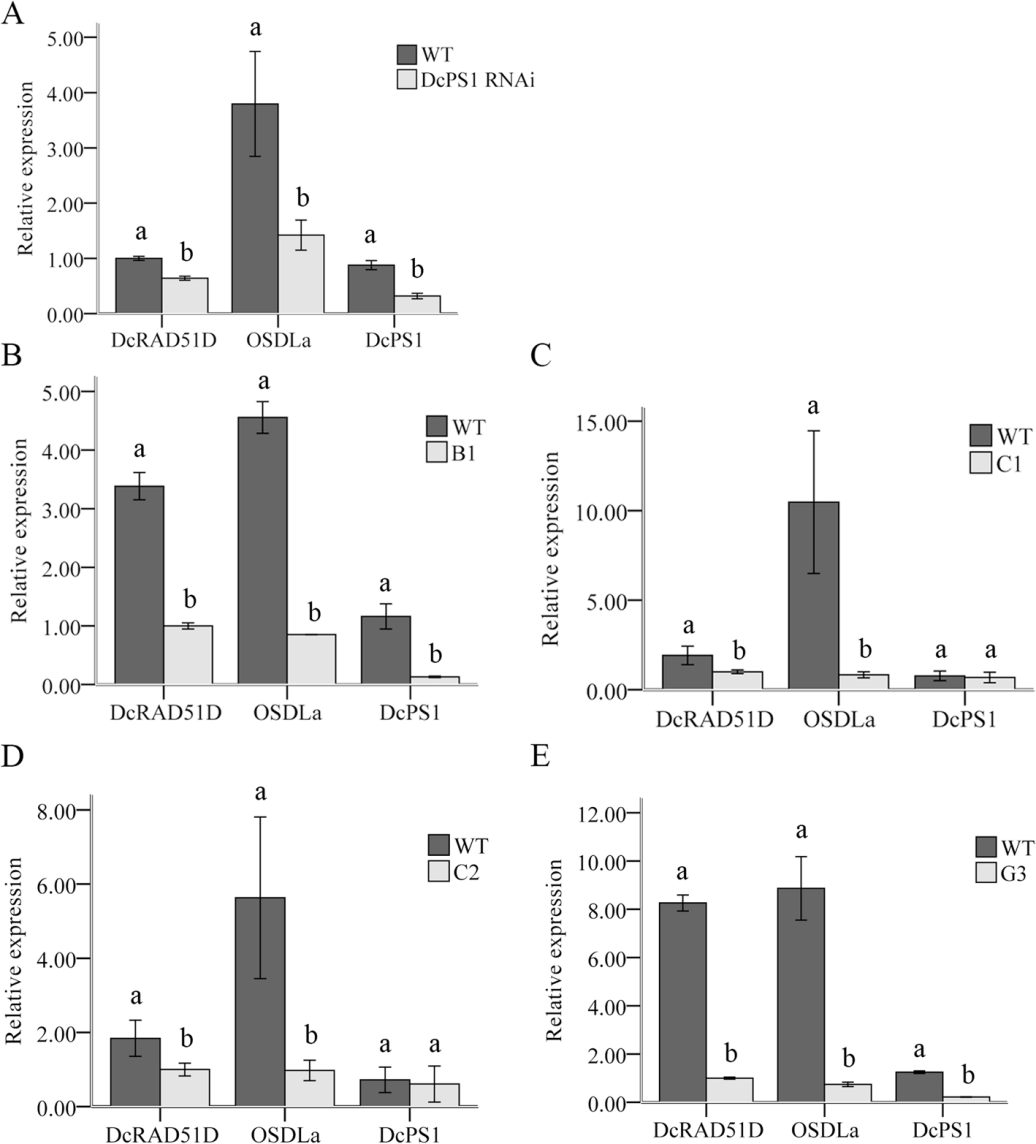
Differential expression of *DcRAD51D*, *OSDLa* and *DcPS1* in the wild type and corresponding DcPS1 RNAi line and OSDLa RNAi line. For quantitative expression analysis, RT-qPCR was performed on RNA harvested from young leaf. (**A**) Transcript levels of *DcRAD51D*, *OSDL1a* and *DcPS1* were quantified in the wild type and corresponding DcPS1 RNAi line. (**B**-**E**) Transcript levels of *DcRAD51D*, *OSDL1a* and *DcPS1* were quantified in the wild type and corresponding OSDLa RNAi plant B1, C1, C2, G3. Glyceraldehyde 3-phosphate dehydrogenase (GAPDH) was used to normalize initial cDNA concentrations. Comparative statistics of the means were checked using a oneway ANOVA test (SPSS 18.0)

To analyse the potential regulatory effects of the DcPS1 protein on *OSDLa* and *DcRAD51D* gene expression and the OSDLa protein on *DcPS1* and *DcRAD51D* gene expression, RT–qPCR analyses using gene-specific primers were performed on RNA harvested from both DcPS1 and OSDLa RNAi lines. In the DcPS1 RNAi lines, a decrease in *OSDLa* and *DcRAD51D* expression was observed (Fig. 6A), indicating that *DcPS1* either positively regulates the expression of *OSDLa* and *DcRAD51D* or prevents the degradation of the corresponding transcript. Compared with the wild type, DcPS1 RNAi lines showed a significant decrease in *DcPS1* gene expression (Fig. 6A), which led to a larger increase in the frequency of dyad and triad formation (Fig. 1K), similar to that in *OSDLa*-RNAi lines (Fig. 6B-E, Fig. 4K). In the OSDLa RNAi lines C1 and C2,there was no significant decrease in *DcPS1* gene expression (Fig. 6C, D) with a smaller increase in the frequency of dyad and triad formation than in the WT (Fig. 4K). However, in the OSDLa RNAi lines B1 and G3, there was a significant decrease in *DcPS1* gene expression (Fig. 6B, E) and a greater increase in the frequency of dyad and triad formation than in the WT (Fig. 4K). Because the penetrance of the 2n pollen phenotype (dyad and triad formation) for all lines was reflected by the decrease in *DcPS1* expression, there appeared to be a close correlation between the *DcPS1* expression decrease and the frequency of 2n formation. In *OSDLa* RNAi lines, *DcRAD51D* gene expression was decreased compared to the wild type (Fig. 6), and the Y2H assay and BiFC analysis also confirmed that OSDLa interacted with *DcRAD51D*, which suggested a meiosis-specific function of *OSDLa* in regulating *DcRAD51D* expression.

## Discussion

In this study, we used RNAi to identify and describe the *DcPS1* and *OSDLa* genes that produce increased levels of diploid pollen grains and lead to meiotic defects. We used cytological analyses to carry out an investigation of the mechanism responsible for these 2n pollen grains in *DcPS1* and *OSDLa* and established that they resulted from an absence of the second meiotic division and abnormal orientation of spindles at meiosis II. Interestingly, defects in meiosis II spindles in *atps1* mutants, which did skip the second meiotic division, are responsible for the formation of 2n spores [5]. However, *osd1* mutants in *Arabidopsis* produce diploid gametes by skipping the second meiotic division, but do not show abnormal orientation of the spindles [21]. The *DcPS1* and *OSDLa* genes are therefore a good candidate genes for producing 2n pollen, which is extensively used in carnation breeding programmes.

DcPS1 protein contains two highly conserved domains, a forkhead-associated domain (FHA) and a C-terminal PilT N-terminal domain (PINc) [27]. The FHA domain is conserved in most homologous domains within the N-terminal domain, and its motif usually has a well-known phosphorylation protein recognition function,which is essential for the DNA damage-related signalling pathway and cell cycle progression, similar to Rad53-Rad9 in budding yeast [31–35]. The PINc domain is predicted to have RNA-binding properties and is usually associated with RNA nuclease activity [36]. In eukaryotes, PINc-containing proteins, such as SMG6 protein families, are related to nonsense-mediated mRNA decay (NMD), which recognizes and rapidly degrades mRNAs so that translation is terminated in advance [37–39]. Therefore, several proteins containing the PINc domain are involved in RNAi, RNA maturation, or RNA decay [5, 40, 41].

**Fig. 7:**
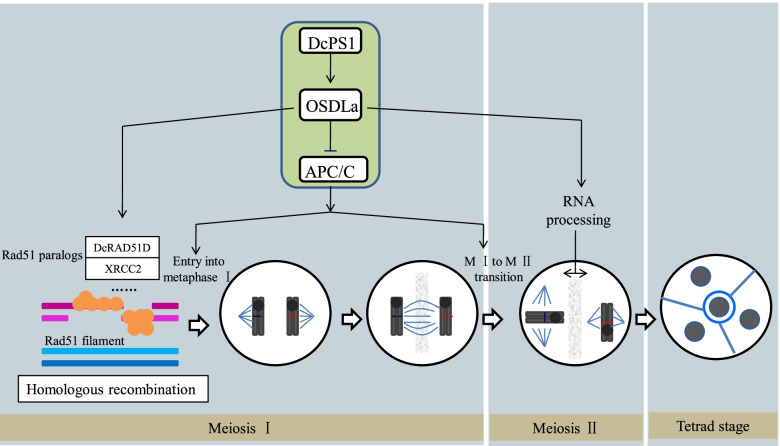
A simplified model for the *DcPS1* involved in diploid gamete formation in carnation

*DcPS1* plays a key role in diploid gametogenesis. Figure 7 shows the involvement of *DcPS1* in meiosis process control in carnation. Arabidopsis *RAD51* paralogues *XRCC2*, *RAD51B* and *RAD51D* have been found to play a role in recombination in the RAD51-independent single-strand annealing pathway [42]. *RAD51D* contains a functional Walker A and B ATPase motif that interacts with *XRCC2* and *RAD51C* and for efficient homologous recombinational repair [43, 44]. Proteins containing the FHA domain have been described to respond to signals related to DNA replication and repair. The function of the FHA domain in regulatory pathways is related to Ser/Thr phosphorylation [45], and this domain appears to be a modular protein-binding domain [32, 35]. Therefore, *DcPS1* containing the FHA domain regulates *OSDLa* and *DcRAD51D* and may participate in homologous recombinational repair in carnation.

DcPS1 RNAi lines generate restored dyads to produce 2n gametes under an absence of the second meiotic division. A second protein essential for entry into M II is *OSDLa*. Loss of function of *OSDLa* in carnation induces skipping of M II, thus generating 2n gametes. *DcPS1* most presumably appears to interact with *OSDLa* to regulate meiotic cell cycle progression, promoting entry into M II (Fig. 7). Reduction in *DcPS1*and *OSDLa* expression levels also resulted in inducing tripolar spindles in male M II, thus generating triads to produce 2n gametes (Fig. 7). PINc domains have predicted RNA-binding properties and are generally found in proteins involved in RNA processing and nonsense-mediated RNAdecay. As such, *DcPS1* has been speculated to interact with *OSDLa* and play a regulatory role likely via NMD or RNA processing, which may control the orientation of M II spindles .

We additionally show that the reduction in *DcRAD51D* and *OSDLa* expression levels is caused by a reduction in *DcPS1* transcript levels, indicating that *DcPS1* positively regulates *DcRAD51D* and *OSDLa* expression, allowing homologous recombinational repair, entry into M II and the proper orientation of metaphase II spindle plates in carnation.

Further studies involving DcPS1 should be helpful to further reveal the mechanism of meiosis in carnation. The isolation of 2n gametogenesis-related genes is important for deciphering the meiosis mechanism and may also have potential basic applications in evolutionary studies and plant breeding programmes.

## Conclusions

We conclude that *DcPS1* plays a role in meiosis I as well as in meiosis II and that *DcPS1* and *OSDLa* are part of a network that links homologous recombination, the proper orientation of the metaphase II spindle and entry into meiosis II. Our results highlight the importance of *DcPS1* and *OSDLa* for cell cycle progression.

## Supplementary Information

**Additional file 1.**

**Additional file 2.**

## Data Availability

The datasets generated during the current study are available in the NCBI, *DcPS1* (GenBank number KR013247), *OSDLa* (GenBank number KX622764) and *DcRAD51D* (GenBank number MK733915).
