# Supplementary material for: The DcPS1 cooperates with OSDLa during pollen development and 2n gamete production in carnation meiosis
Source: BMC Plant Biol. 2022 May 24;22:259. doi: 10.1186/s12870-022-03648-z (PMC9128087; doi:10.1186/s12870-022-03648-z)
Supplement: Supplementary file 1 — Additional file 1. [file 12870_2022_3648_MOESM1_ESM.pdf]

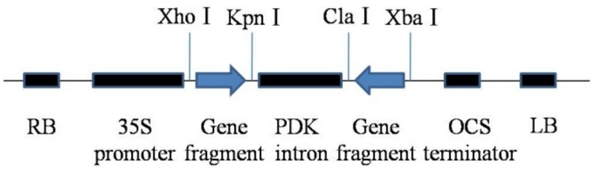

Fig. S1 Schematic representation of RNAi vector used for carnation transformation. RB, right border; Gene fragment, *DcPS1* or *OSDLa* gene fragment of carnation; LB, left border.

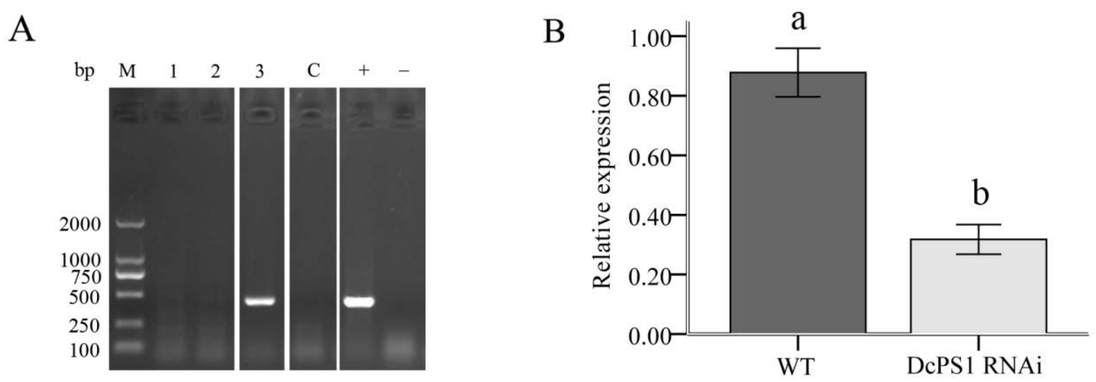

Fig. S2 Molecular identification of the transgenic carnation lines of DcPS1 RNAi. (A) PCR identification of transgenic lines. M, DNA marker; 1-2, transgenic negative plant; 3, transgenic positive plant; +, positive control; –, no template control; C untransformed control. The grouping of gels cropped from different parts of the same gel using white space. (B) qRT-PCR results of *DcPS1* expression in selected transgenic lines. DcPS1 RNAi lines show a significant reduction ($P < 0.05$) in *DcPS1* RNA levels compared with the wild type (WT).

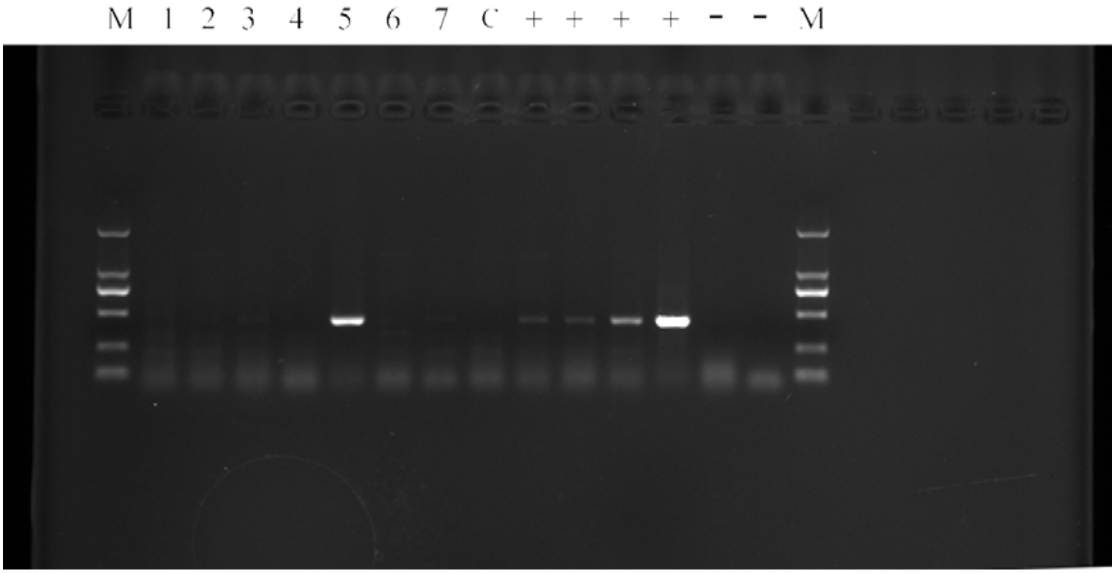

Fig. S3 Full-length gel of PCR identification for supplementary information of figure S2A. M, DNA marker; 1-7, transgenic plant; +, positive control; –, no template control; C, untransformed control.

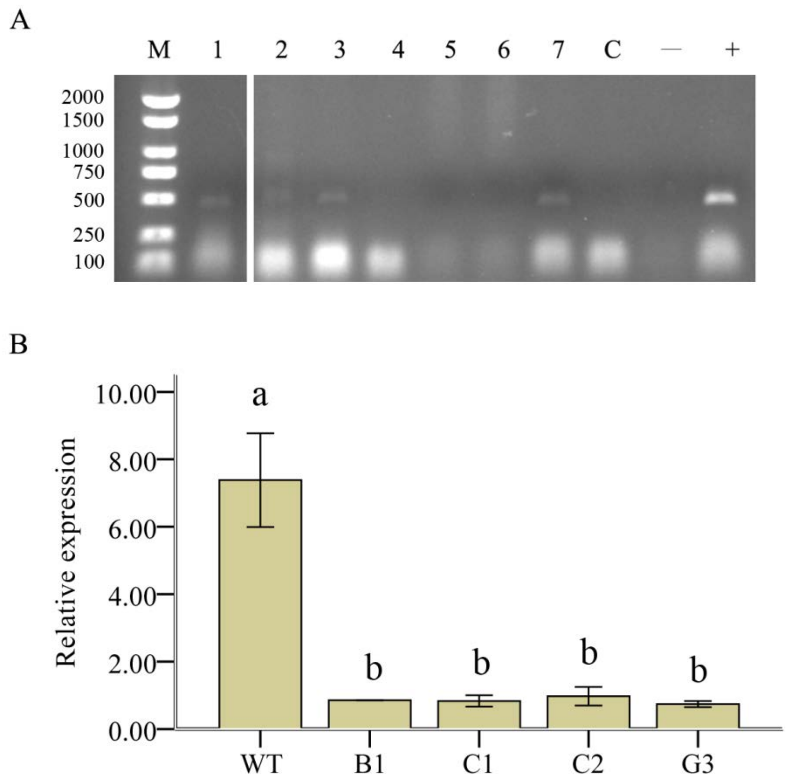

Fig. S4 Molecular identification of the transgenic carnation lines of *OSDLa*. (A) PCR identification of transgenic lines. Line 4-6 were scored as negative for transformation. Lines 1-3,7 were scored as positive for transformation.

M, DNA marker; +, positive control; –, no template control; C, untransformed control. The grouping of gels cropped from different parts of the same gel using white space. (B) OSDLa RNAi lines show a significant reduction in *OSDLa* expression levels by qRT-PCR ($P < 0.05$), C1, G3, B1, and C2, transgenic positive plants.

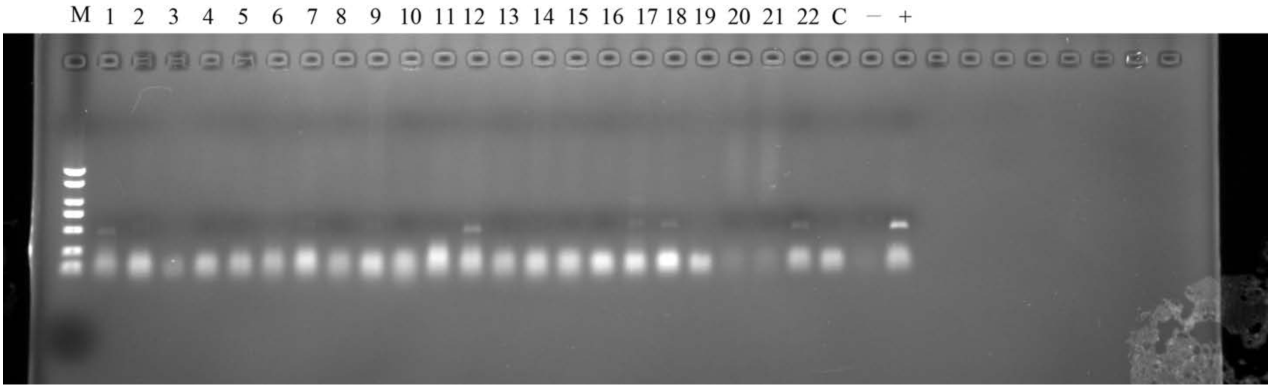

Fig. S5 Full-length gel of PCR identification for supplementary information of figure S4A. M, DNA marker; 1-22, transgenic plant; +, positive control; –, no template control; C, untransformed control.

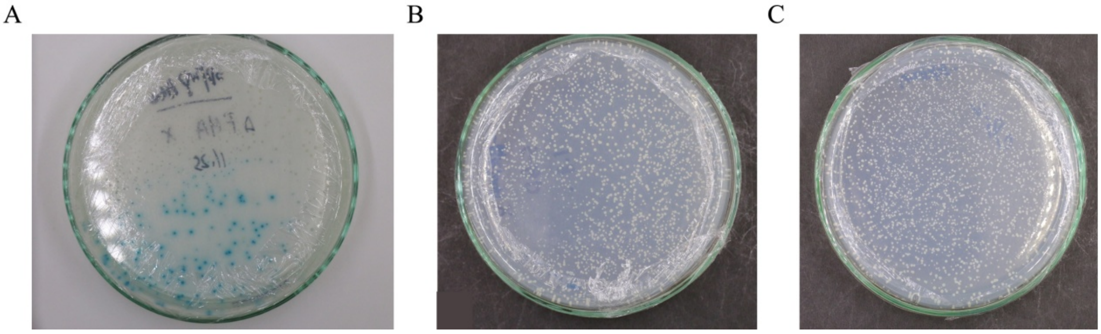

Figure S6 Confirmation of bait autoactivation by β-galactosidase assay. The Y2H yeast cells were transformed with the pGBKT7-DcPS1 vectors (A), pGBKT7-DcRAD51D vectors (B) and pGBKT7-OSDLa vectors (C) for the autoactivation test. The yeast transfected with plasmids pGBKT7-DcRAD51D vectors (B) and pGBKT7-OSDLa vectors (C) did not turn blue in the β-galactosidase assay, but pGBKT7-DcPS1 vectors turn blue.
