# Supplementary material for: The DcPS1 cooperates with OSDLa during pollen development and 2n gamete production in carnation meiosis
Source: BMC Plant Biol. 2022 May 24;22:259. doi: 10.1186/s12870-022-03648-z (PMC9128087; doi:10.1186/s12870-022-03648-z)
Supplement: Supplementary file 2 — Additional file 2. [file 12870_2022_3648_MOESM2_ESM.pdf]

Table S1 Primers used in this study. FP: forward primer, RP: reverse primer

Name	Primer sequence 5' to 3'
PDK-1262F	FP GATTACAGTTGGGAAATTGGGT
DcPS1 R	RP CGTTGCCAAATGGTTATCTG
PDK F	FP GTTAGAAATTCCAATCTGCTTGTAA
OSDLa R	RP ACGGTACTTGGCTAGGTTGC
GAPDH 579-F	FP CAGTGGGCACACGGAAAGC
GAPDH 788-R	RP TGGCATCGTTGAGGGTCT
OSDLa-422F	FP CGGAGTGTCTTACCTTCTT
OSDLa-656R	RP CTCTCGCAATCTCGTCTT
DcPS1-2118F	FP AAAACTGGATGGTGGATTCA
DcPS1-2225R	RP AAAGCCTGAGAACGAGTATGA
DcRAD51D-530F	FP TCACGGTGGTTTACGAGAAGGGTTT
DcRAD51D-701R	RP GATAAGGCGAGCAATGCGGGATG
DcPS1 F	FP AGCAGAAGCTGATCTCAGAGGAGGACCT GCATATGATGTCGAAACTCCAAAAAGAGAGA
DcPS1 R	RP GCAGGTCGACGGATCCCCGGGAATTCTT AGGCTGCTGCGTTCACTCGTTC
OSDLa F	FP AGCAGAAGCTGATCTCAGAGGAGGACCT GCATATGATGCCGGTATCCAGGGA
OSDLa R	RP GCAGGTCGACGGATCCCCGGGAATTCTC ATCGCATCGACATCAATG
DcRAD51D F	FP AGCAGAAGCTGATCTCAGAGGAGGACCT GCATATGATGGTTTTGCTGAAAACGATG
DcRAD51D R	RP GCAGGTCGACGGATCCCCGGGAATTCT TAGGAAGAAGATTGTGACCAAC
